# The FEDRA Longitudinal Study: Repeated Volumetric Breast Density Measures and Breast Cancer Risk

**DOI:** 10.3390/cancers15061810

**Published:** 2023-03-16

**Authors:** Giovanna Masala, Melania Assedi, Benedetta Bendinelli, Elisa Pastore, Maria Antonietta Gilio, Vincenzo Mazzalupo, Andrea Querci, Miriam Fontana, Giacomo Duroni, Luigi Facchini, Calogero Saieva, Domenico Palli, Daniela Ambrogetti, Saverio Caini

**Affiliations:** 1 Clinical Epidemiology Unit, Institute for Cancer Research, Prevention and Clinical Network (ISPRO), 50139 Florence, Italy; 2 Cancer Risk Factors and Lifestyle Epidemiology Unit, Institute for Cancer Research, Prevention and Clinical Network (ISPRO), 50139 Florence, Italy; 3 Breast Cancer Screening Branch, Institute for Cancer Research, Prevention and Clinical Network (ISPRO), 50139 Florence, Italy

**Keywords:** volumetric mammographic breast density, breast cancer risk, digital mammograms, repeated measures, longitudinal study

## Abstract

**Simple Summary:**

Mammographic breast density is a strong independent risk factor for breast cancer. We investigated the association between volumetric mammographic breast density measures, their changes over time, and breast cancer risk in a cohort of women participating in the FEDRA (Florence-EPIC Digital mammographic density and breast cancer Risk Assessment) study. For the 6148 study women (262 breast cancer cases, average 7.8 years of follow-up), repeated measures of mammographic breast density from full-field digital mammograms and repeated information on lifestyle habits, reproductive history, and anthropometry were available. The association between mammographic breast density measures (modeled as time-dependent covariates), their relative annual changes, and breast cancer risk was evaluated by adjusted Cox models. Higher values of volumetric percent density and dense volume were positively associated with increased breast cancer risk, while an inverse association was evident for increasing non-dense volume. No clear effect of annual changes in mammographic breast density emerged.

**Abstract:**

Mammographic breast density (MBD) is a strong independent risk factor for breast cancer (BC). We investigated the association between volumetric MBD measures, their changes over time, and BC risk in a cohort of women participating in the FEDRA (Florence-EPIC Digital mammographic density and breast cancer Risk Assessment) study. The study was carried out among 6148 women with repeated MBD measures from full-field digital mammograms and repeated information on lifestyle habits, reproductive history, and anthropometry. The association between MBD measures (modeled as time-dependent covariates), their relative annual changes, and BC risk were evaluated by adjusted Cox models. During an average of 7.8 years of follow-up, 262 BC cases were identified. BC risk was directly associated with standard deviation increments of volumetric percent density (VPD, HR 1.37, 95%CI 1.22–1.54) and dense volume (DV, HR 1.29, 95%CI 1.18–1.41). An inverse association emerged with non-dense volume (NDV, HR 0.82, 95%CI 0.69–0.98). No significant associations emerged between annual changes in VPD, DV, NDV, and BC risk. Higher values of MBD measures, modeled as time-dependent covariates, were positively associated with increased BC risk, while an inverse association was evident for increasing NDV. No effect of annual changes in MBD emerged.

## 1. Introduction

Breast cancer (BC) is the most commonly diagnosed cancer and the leading cause of cancer death among women worldwide and in all European countries. In Europe, the cumulative risk of a BC diagnosis by the age of 75 is 8% (1 in 12 women), while the risk of BC-associated death before the age of 75 is 1.6% (1 in 61 women) [1,2].

Mammographic breast density (MBD) refers to the proportion of fibroglandular tissue (radio-dense in mammograms) in relation to total breast volume. MBD can be modulated by known BC risk factors [3]. Reproductive and hormonal factors [4,5,6,7], diet, and physical activity (PA) [8,9,10,11,12,13,14,15,16,17,18] seem to act in a similar way in modulating MBD and BC risk. Parity, early age at first birth, consumption of vegetables, intake of antioxidants, and increased PA are reported to be inversely associated with both MBD and BC risk. Hormone replacement therapy and intakes of protein, saturated fat, and alcohol are reported to be directly associated with both MBD and BC risk. On the other hand, age and body mass index (BMI) are inversely associated with MBD but positively associated with BC risk [3].

MBD is a strong independent risk factor for BC and several studies have shown that women with high MBD have a two- to six-fold increase in BC risk compared with women with low MBD [19,20]. Most of these studies used film-screen mammography and reader-dependent qualitative categorisation of MBD such as BI-RADS scoring system or computer-assisted mammographic density assessment [21,22,23]. Over the last decade, full-field digital mammography (FFDM) has progressively replaced film-screen mammography in screening programmes and the development of automated programs for volumetric MBD assessment allowed us to obtain volumetric MBD measures. Some studies reported the performances of automated quantitative assessment programs in the prediction of BC risk and the associations with BC risk factors [24,25]. Studies exploring the association of MD with BC risk were mainly based on a single measure of density with a large variation in the time distance between BC occurrence and the negative mammogram used to assess MBD. The availability of standardised and highly reproducible volumetric breast density measures can support studies aimed to evaluate the effect of MBD on BC over time and the role of MBD changes in BC risk prediction [26].

We present in this paper the results of a study aimed to prospectively investigate the association between volumetric MBD assessed through an automated density assessment software and BC risk. The analysis was carried out in a cohort of women with repeat volumetric MBD measures and extensive information on known BC risk factors, participating in the FEDRA (Florence-EPIC Digital mammographic density and breast cancer Risk Assessment) study.

## 2. Materials and Methods

### 2.1. Study Population

The FEDRA longitudinal study was carried out among the women, previously enrolled in the European Prospective Investigation into Cancer and Nutrition (EPIC) Florence cohort (1993–1998), with at least an FFDM performed after the enrollment in the original cohort and for which quantitative MBD measurements were available (n. 6148). Women included in the FEDRA longitudinal study were on average younger at enrollment in the EPIC study as compared to women without FFDMs. The latter, given their older age at enrollment, in 2004 (when FFDM started being used in the local programme) were out of the age range of BC screening. The FEDRA study was approved by the local Ethics Committee “Toscana Area Vasta Centro”. Participants signed a specific informed consent form. All procedures performed were in accordance with the ethical standards of the institutional and national research committees and with the 1964 Helsinki declaration and its later amendments or comparable ethical standards.

### 2.2. Mammographic Examination Retrieval and Breast Density Assessment

The mammographic examination history of all participants was obtained through a linkage with the archives of the local population-based mammographic screening programme (performed at ISPRO, the Regional Health System Institution in charge of the mammographic screening in the Florence area). In the frame of the FEDRA study, mammographic examinations of each participant have been identified (until 31 December 2021) including all FFDMs progressively implemented in the local BC screening programme since 2004 (with full coverage from 2011 onwards).

For the available FFDMs, MBD measures were obtained. The fully automated Volpara^TM^ density software (version 3.1, Matakina Technology, Wellington, New Zealand) was used to determine quantitative measures of MBD, including total breast volume (cm^3^), absolute breast dense volume (DV, cm^3^) and volumetric percent density (VPD, %), from raw (“for processing”) FFDMs images. The technical characteristics of the Volpara system have been already described in detail [27]. Briefly, the algorithm computes the thickness of dense tissue at each pixel using the X-ray attenuation of an entirely fatty region as an internal reference. The thickness values over the whole breast region are integrated to obtain the absolute DV (cm^3^). Total breast volume (cm^3^) is then obtained by multiplying the breast area by the recorded breast thickness, corrected for the breast edge. Non-dense volume (NDV, cm^3^) is derived as the difference between total breast volume and DV. VPD is then obtained from the ratio of DV and total breast volume. In the present study, we used the average MBD values obtained from mediolateral oblique and craniocaudal views of the right and left breasts.

The Volpara^TM^ density software also provides a Volpara Density Grade (VDG 5th edition) which correlates with the breast density categories of ACR BI-RADS 5th edition. The VDG is based on VPD, which is divided as follows: 0.0–4.5% (VDG1 corresponding to BI-RADS 1), 4.5–7.5% (VDG2 corresponding to BI-RADS 2), 7.5–15.5% (VDG3 corresponding to BI-RADS 3), and ≥15.5% (VDG4 corresponding to BI-RADS 4) [28,29].

### 2.3. Follow-Up and BC Cases Ascertainment

The ascertainment of vital status was carried out through the linkage with the local town offices and the local mortality registries, thereby identifying the deceased subjects and the date and cause of death. The identification of BC cases (invasive and in situ epithelial cancers) was obtained through linkages with the hospital discharge system, the population-based Cancer Registry (active in the Florence area since 1985), other sources such as Pathology Department registries (BC was coded as C50 according to ICD-O-2 classification) and active follow-up through participants. Follow-up was closed on 31 December 2019 both for vital status and BC incidence. For the present analysis, BC cases diagnosed after the first FFDM till 31 December 2019 were considered.

### 2.4. Covariates

As a part of the EPIC Florence cohort, every woman included in the FEDRA study provided detailed information at enrollment on educational level, reproductive history (parity, number of children alive, breastfeeding, age at menarche, age at menopause), medical history including menopausal hormone therapy, smoking and alcohol drinking history, physical activity habits and dietary information. Anthropometric measures were also collected by trained nurses according to an international standard protocol [30]. An update of the information on lifestyle, medical history, reproductive history, and anthropometric measures was carried out in 2004–2005 (after a 9.4-year average follow-up) when participants were invited by mail to complete a self-administered questionnaire and to provide self-measured weight, hip and waist circumferences following specific instructions and using a measuring tape supplied by the study center [31], and then again after 15 years of follow up in the frame of the FEDRA study when an update of dietary information was also obtained.

### 2.5. Statistical Analysis

Descriptive statistics of the main baseline characteristics of the study women (distribution and percentages for categorical variables, means and standard deviations for continuous variables) were calculated, separately for BC cases and non-cases. BMI was calculated as weight (kg) over squared height (m^2^), and women were categorised as normal weight (BMI < 25 kg/m^2^), overweight (25 kg/m^2^ ≥ BMI < 30 kg/m^2^), or obese (BMI ≥ 30 kg/m^2^). For each participant available information on age at first birth, the number of births, and time between births were summarised by means of the Birth Index. Birth Index was calculated as age at EPIC enrollment or age at menopause (whichever comes first) minus age at every birth. The birth index was set to 0 for nulliparous women. A higher Birth Index indicates a higher number of births occurring at earlier ages [32]. Means, standard deviations, and *p*-values from general linear models were performed for continuous variables. Frequencies and Pearson’s chi-squared tests were performed for categorical variables.

The association between BC risk and the MBD measures (VPD, DV, and NDV) were evaluated by means of separate Cox proportional hazards models. Hazard ratios (HR) and 95% confidence intervals (CI) were estimated. Age was used as the primary time variable. Women were considered at risk from the time of the first FFDM until BC diagnosis or censoring (death for other causes, loss to follow-up, end of follow-up, whichever came first). The peculiarity of this analysis is the availability, for study subjects, of repeated MBD measures that have been modeled as time-dependent covariates [33].

MBD measures (VPD, DV, and NDV) were analyzed as continuous standardised variables, obtained by subtracting the mean value of each measure from the value of each woman and then dividing by the standard deviation. DV and NDV were also added as dummy variables of quartiles (with the lowest quartile as the reference category). The linearity of trends across quartiles was tested by treating quartiles as a continuous variable. VPD was also added as dummy variables of VDG categories (VDG1 category as reference). The linearity of trends across VDG categories was tested by treating VDG categories as a continuous variable.

Cox models were adjusted for age at FFDM (continuous), BMI (continuous), alcohol intake (never drinkers, ex-drinkers, current drinkers of ≤1, ≤2, ≤3, ≤4, and >4 alcohol units), Birth Index and menopausal hormone therapy (as time-dependent covariates choosing each time the value closest to the consecutive FFDMs), educational level, and age at menarche.

For each woman with consecutive FFDMs, we calculated the annual changes in VPD, DV, and NDV as secondary outcomes of the study. For two consecutive FFDMs at age1 and age2, we defined the VPD change per year as (VPD2-VPD1)/VPD1/(age2–age1). The same procedure was applied to DV and NDV changes per year. Annual change in VPD, DV, and NDV was then categorised as decrease (annual decrease > 10%), increase (annual increase > 10%), or stable (annual change ≤ 10%). As a sensitivity analysis, we also performed analysis considering a 5% cut-off for the categorisation of annual change in VPD, DV, and NDV.

Cox proportional hazards models were fitted to evaluate the association between BC risk and annual changes categories in VPD, DV, and NDV, respectively (with the “stable” category as a reference). Annual change categories were modeled as time-dependent covariates. Cox models were adjusted as previously reported.

Analyses were carried out using SAS Statistical Package 9.2 (SAS Institute Inc., Cary, NC, USA).

## 3. Results

This analysis refers to 6148 women who had previously joined the EPIC Florence cohort for which at least one FFDM was identified and MDB measures obtained by applying the Volpara^TM^ software version 3.1.

Characteristics at first FFDM of the 6148 women included in the FEDRA study, overall and separated by BC status are reported in Table 1. During a mean follow-up of 8.7 years (from the first FFDM to 31 December 2019), a total of 262 women were diagnosed with BC (212 invasive and 50 in situ epithelial cancers). Later age at first birth, lower number of children, lower breastfeeding duration, higher BMI and current alcohol consumption were slightly more frequent among BC cases than among non-cases. No differences emerged in age at first FFDM and age at menarche between BC cases and non-cases. The mean number of FFDMs per woman was 3.1 (SD 1.5) with 1.8 years of mean intervals between consecutive FFDMs and a mean distance of 6.6 years between the first and last FFDM. The mean number of FFDMs was higher among non-cases (3.1; SD 1.5) than among BC cases (2.2; SD 1.3). At first FFDM (the one that determined the inclusion in the study) the average VPD was 7.6 (SD 4.9) among non-cases and 8.7 (SD 5.1) among cases. The average DV was 40.0 cm^3^ (SD 25.0) among non-cases and 55.3 cm^3^ (SD 28.1) among cases. The average NDV was 730.1 cm^3^ (SD 396.4) among non-cases and 693.6 cm^3^ (SD 366.7) among cases. VPD, DV, and NDV values distributions in consecutive FFDMs by BC status are reported in Figure S1, Figure S2 and Figure S3, respectively.

Fully adjusted Cox proportional hazards models showed a significantly higher BC risk associated with higher values of VPD and DV. These associations were evident considering standard deviation increments (VPD: HR 1.37, 95%CI 1.22–1.54; DV: HR 1.29, 95%CI 1.18–1.41), VDG categories (HR 3.57; 95%CI 2.08–6.12 VDG4 vs. VDG1; *p* trend <0.0001) and quartiles of DV (HR 2.30; 95%CI 1.63–3.26 highest vs. lowest quartile; *p* trend <0.0001). Higher values of NDV were significantly associated with a lower BC risk (HR 0.82, 95%CI 0.69–0.98) (Table 2).

Overall, weak and not significant associations emerged between annual changes in VPD and NDV and BC risk. Compared to women with stable VPD, BC risk was reduced by over 30% among women with 10% decreased VPD (HR 0.68, 95%CI 0.40–1.16), and increased by slightly over 20% among women with 10% increased VPD (HR 1.21, 95%CI 0.81–1.82), although statistical significance was not achieved in either comparison. BC risk was also higher among women with 10% decreased NDV (HR 1.37, 95%CI 0.84–2.23) and lower among women with 10% increased NDV (HR 0.67, 95%CI 0.41–1.11) compared with stable women, although not achieving statistical significance (Table 3). Analysis with the 5% cut-off in annual changes in VPD and NDV made little difference to the results.

## 4. Discussion

We studied the longitudinal association between repeated quantitative measurements of mammographic breast density parameters and breast cancer risk in a cohort of 6148 women with at least one FFDM obtained in the local population-based breast cancer screening programme. In this cohort, we identified 262 newly diagnosed BC during a median follow-up of 8.7 years. Upon adjusting for several potential confounders (including a woman’s age, menstrual and reproductive history, use of exogenous sex hormones, anthropometry, diet, and alcohol intake), we found that higher values of volumetric percent density and absolute dense volume (entered in the analyses as time-dependent covariates) were positively associated with increased risk of subsequent BC, with a clear dose-response relationship, while an inverse association with BC risk was suggested for increasing volumes of non-dense breast tissue.

The findings from the present investigation are largely consistent with previous evidence on the topic and add to the notion that women with dense breasts are at increased risk of developing BC during their lifetime. The earliest studies reporting a link between higher MBD and increased BC risk, including seminal works by Boyd et al. [20], McCormack et al. [19], and others, were based on film-screen mammograms assessed by radiologists using reader-dependent tools. In a previous investigation conducted within the EPIC-Florence cohort (a nested case-control study encompassing 136 BC cases and 635 controls), we found a more than 2.5 times increased BC risk among women falling in the BI-RADS category 4 vs. 1 (corresponding to fibroglandular tissue accounting for >75% vs. <25% of total breast volume) [34]. In recent years, FFDM has progressively replaced the use of analog mammography, however, the number of studies that examined longitudinally (i.e., by taking advantage of repeated measurements) the link between software-assessed MBD (and changes thereof over time) and BC risk is still limited, and their findings were rather inconsistent. Moreover, they are mostly based on area-based measures of MBD whereas the Volpara^TM^ density software performs a volumetric measure of breast density, thus making it difficult to compare the results. In an early case-control study conducted by van Gils et al. in Nijmegen, Netherlands, including 108 post-menopausal BC cases and 400 controls, BC risk was significantly increased (OR 6.9) among women whose percent MBD increased over time from <5% to 5–25% compared to those with <5% density throughout the whole study period [35]. Moreover, women whose initial percent MBD was in the 5–25% range had a reduced BC risk if their percent MBD fell into the <5% category compared to if it stayed constant over time. In contrast, taking into consideration changes in area-based percent MBD over time did not improve the ability to predict BC development compared to when only the first available percent MBD value was considered, in the case-control study conducted in the US (Mayo Clinic) by Vachon and colleagues and encompassing 372 BC cases and 713 matched controls [36]. Lokate et al. conducted a case-control study (533 BC cases and 1367 controls) nested within the EPIC-Netherlands cohort and found suggestive indications that large increases in area-based percent MBD over time may be associated with increased BC risk [37]. Work et colleagues set up a case-control study nested in a cohort of high-risk women in the US, and found that the annual change in area-based percent MBD (determined by means of the Cumulus software) between two consecutive digital mammograms was +0.29% vs. −1.62% among BC cases and age-matched controls, respectively, the difference being statistically significant (*p*-value < 0.009) [38]. Finally, Azam et al. reported that area-based MBD changes over time neither represented a BC risk factor per se nor influenced the association between baseline MBD and subsequent BC risk, in a large cohort of Swedish women (the KARMA study, *n* = 48,310, of which 563 developed BC during follow-up) whose mammograms were examined using the STRATUS fully automated tool [26]. By and large, most of the still few studies published to date, including the present one, found that longitudinal changes in VPD may significantly affect BC risk, although null results were reported by a few studies and more research is therefore needed.

Our results largely corroborate previous investigations conducted within the same cohort as well as independent reports from other research groups, and may also help enlighten the ongoing debate about whether (and how) BC screening modalities should be tailored for women with dense breasts based on the breast composition parameters measured at the first mammogram.

Our study has some strengths that it is important to emphasise as they jointly contribute to heightening the scientific soundness and reliability of its findings. The FEDRA study is nested within a well-characterised general population-based cohort with plenty of information available on potential confounders of the association being studied. FFDMs were obtained within a BC screening programme that has been in place for decades, and the use of automated software for the quantification of breast composition parameters ensures the validity and reproducibility of exposure measures. Our study is longitudinal in nature thanks to the availability of an average of over three digital mammograms per woman (which was made possible by the long follow-up of the EPIC-Florence cohort) and the use of statistical methods able to accommodate time-varying exposures. Our study has also some limitations that deserve to be fully acknowledged. Unlike breast tissue composition parameters (i.e., VPD, DV, and NDV), repeated information on potential confounders of the association under study is currently limited or lacking (e.g., for dietary habits), which may have curbed our ability to remove confounding. In addition, by being based on women who volunteered to join the EPIC study and mostly regularly participate in BC screening, some selection bias (e.g., healthy participant/user bias) is likely to have been at play.

## 5. Conclusions

In conclusion, we confirmed that MBD is longitudinally associated with a substantial increase in the risk of developing BC over the lifetime among women from the general population attending the BC screening programme. This result was obtained taking into account a large series of potential confounders and was confirmed for both relative and absolute measures of breast density. No clear evidence of an effect of mammographic breast density changes on BC risk in the time interval considered emerged.

## Figures and Tables

**Table 1:** Main characteristics of the 6148 women included in the FEDRA study separated by breast cancer status.

Characteristics	Total Women	Breast Cancer	
	(*n* = 6148)	Yes (*n* = 262)	No (*n* = 5886)
Age at first FFDM (years)			
Mean (SD)	64.1 (6.6)	64.3 (6.8)	64.1 (6.6)
Mean (10°–90°)	65.0 (54.0–72.0)	65.0 (55.0–72.0)	65.0 (54.0–72.0)
Number of consecutive FFDM			
Mean (SD)	3.1 (1.5)	2.2 (1.3)	3.1 (1.5)
Mean (10°–90°)	3.0 (1.0–5.0)	2.0 (1.0–6.0)	3.0 (1.0–5.0)
Volumetric percent density (%)			
Mean (SD)	7.6 (4.9)	8.7 (5.1)	7.6 (4.9)
Mean (10°–90°)	6.1 (3.1–14.1)	7.5 (3.7–15.4)	6.1 (3.1–14.1)
Dense volume (cm^3^)			
Mean (SD)	48.1 (25.1)	55.3 (28.1)	48.0 (25.0)
Mean (10°–90°)	42.1 (24.7–77.8)	49.1 (25.5–93.4)	42.0 (24.7–77.6)
Non Dense Volume (cm^3^)			
Mean (SD)	729.6 (396.0)	693.6 (366.7)	730.1 (396.4)
Mean (10°–90°)	659.6 (289.8–1263.3)	627.5 (292.0–1174.2)	659.8 (289.8–1264.6)
Age at menarche			
Mean (SD)	12.4 (1.4)	12.4 (1.4)	12.4 (1.4)
Mean (10–90)	12.0 (11.0–14.0)	12.0 (11.0–14.0)	12.0 (11.0–14.0)
Number of children, N (%)			
0	955 (15.5)	41 (15.6)	914 (15.5)
1–2	4518 (73.5)	199 (75.9)	4319 (73.4)
3+	675 (11.0)	22 (8.4)	653 (11.1)
Breast-feeding duration			
Mean (SD)	6.9 (6.1)	6.2 (5.7)	6.9 (6.1)
Mean (10–90)	6.0 (0.0–15.0)	4.5 (0.0–14.6)	6.0 (0.0–15.0)
Age at first birth			
Mean (SD)	26.5 (4.4)	27.2 (4.7)	26.5 (4.4)
Mean (10–90)	26.0 (21.0–33.0)	27.0 (21.0–33.0)	26.0 (21.0–33.0)
Menopausal status, N (%)			
Post	4944 (80.4)	218 (83.2)	4726 (80.3)
Pre	934 (15.2)	38 (14.5)	896 (15.2)
Peri	182 (3.0)	6 (2.3)	176 (3.0)
Missing	88 (1.4)	-	88 (1.5)
Menopausal hormone therapy, N (%)			
Yes	408 (6.6)	16 (6.1)	392 (6.7)
No	4608 (75.0)	208 (79.4)	4400 (74.7)
Missing	1132 (18.4)	38 (14.5)	1094 (18.6)
Body Mass Index (kg/m^2^)			
Mean (SD)	25.9 (4.4)	26.2 (4.2)	25.9 (4.4)
Mean (10°–90°)	25.2 (21.0–31.7)	25.4 (21.8–31.2)	25.1 (20.9–31.8)
Body Mass Index classes, N (%)			
Underweight/normal weight	2957 (48.5)	119 (45.4)	2838 (48.2)
Overweight	2161 (35.4)	101 (38.6)	2060 (35.0)
Obese	983 (16.1)	42 (16.0)	941 (16.0)
Missing	47 (0.8)	-	47 (0.8)
Alcohol consumption, N (%)			
Former drinkers	381 (6.2)	10 (3.8)	371 (6.3)
Never drinkers	426 (6.9)	19 (7.2)	407 (6.9)
Current drinkers	5341 (86.9)	233 (88.9)	5108 (86.8)
Smoking status N (%)			
Current	1294 (21.0)	45 (17.2)	1249 (21.2)
Former	1998 (32.5)	95 (36.3)	1903 (32.3)
Never	2838 (46.2)	121 (46.2)	2717 (46.2)
Missing	18 (0.3)	1 (0.4)	17 (0.3)

**Table 2:** Hazard ratios (HRs) and 95% confidence intervals (CI) of breast cancer risk in relation to mammographic density measures in the 6148 FEDRA study women. Results from Cox proportional hazards models with repeated mammographic density measures modeled as time-dependent covariates (18,939 total observations: 262 from breast cancer cases and 18,677 from 5886 non-cases).

	N. Cases	HR (95%CI) *	HR (95%CI) **	HR (95%CI) ^#^
Volumetric Percent Density (%)				
Z score	262	1.38 (1.24–1.54)	1.38 (1.23–1.54)	1.37 (1.22–1.54)
VDG1 (mean 3.2; sd 0.6) ^§^	36	1	1	1
VDG2 (mean 5.2; sd 1.1)	89	1.69 (1.13–2.51)	1.68 (1.13–2.50)	1.66 (1.11–2.47)
VDG3 (mean 10.0; sd 2.2)	108	2.99 (1.99–4.50)	2.93 (1.95–4.40)	2.89 (1.92–4.34)
VDG4 (mean 19.7; sd 4.3)	29	3.74 (2.16–6.36)	3.68 (2.14–6.28)	3.57 (2.08–6.12)
*p*-trend		<0.0001	<0.0001	<0.0001
Dense Volume (cm^3^)				
Z score	262	1.30 (1.19–1.43)	1.30 (1.19–1.42)	1.29 (1.18–1.41)
I quartile (min 6.79; max 31.89)	50	1	1	1
II quartile (min 31.90; max 42.10)	47	1.00 (0.67–1.50)	0.97 (0.65–1.45)	0.97 (0.65–1.46)
III quartile (min 42.11; max 57.30)	67	1.47 (1.01–2.13)	1.45 (1.00–2.11)	1.45 (0.99–2.11)
IV quartile (min 57.31; max 307.08)	98	2.37 (1.68–3.34)	2.34 (1.65–3.30)	2.30 (1.63–3.26)
*p*-trend		<0.0001	<0.0001	<0.0001
Non Dense Volume (cm^3^)				
Z score	262	0.81 (0.68–0.97)	0.82 (0.69–0.98)	0.82 (0.69–0.98)
I quartile (min 22.41; max 440.70)	66	1	1	1
II quartile (min 440.71; max 659.50)	72	0.97 (0.68–1.37)	0.98 (0.70–1.39)	0.99 (0.70–1.39)
III quartile (min 659.60; max 947.60)	69	0.83 (0.57–1.21)	0.85 (0.58–1.23)	0.85 (0.58–1.24)
IV quartile (min 947.80; max 3415.2)	55	0.65 (0.41–1.02)	0.66 (0.42–1.04)	0.66 (0.42–1.05)
*p*-trend		0.05	0.06	0.07

z score= (x − µ)/σ. ^§^ VDG = Volpara Density Grade 5th edition (correlated with the breast density categories of ACR BI-RADS 5th edition). * Adjusted for age at FFDM (continuous) and BMI (continuous). ** Adjusted for age at FFDM (continuous), BMI (continuous), alcohol intake (never drinkers, ex-drinkers, current drinkers of ≤1, ≤2, ≤3, ≤4, >4 alcohol units). ^#^ Adjuster for age at FFDM (continuous), BMI (continuous), alcohol intake (never drinkers, ex-drinkers, current drinkers of ≤1, ≤2, ≤3, ≤4, >4 alcohol units), Birth Index, and age at menarche.

**Table 3:** Hazard ratios (HRs) and 95% confidence intervals (CI) of breast cancer risk in relation to changes in mammographic density measures in the FEDRA study (158 breast cancer cases with 2 or more FFDM out of 262; 12,633 repeated FFDM from non-cases out of 18,677).

	N. Cases	HR (95%CI) *	HR (95%CI) **	HR (95%CI) ^#^
Volumetric breast density change per year				
Annual change by more than 10%				
Stable	110	1.00	1.00	1.00
Decrease	16	0.69 (0.40–1.17)	0.68 (0.40–1.17)	0.68 (0.40–1.16)
Increase	32	1.23 (0.82–1.84)	1.22 (0.82–1.83)	1.21 (0.81–1.82)
Dense volume change per year				
Annual change by more than 10%				
Stable	124	1.00	1.00	1.00
Decrease	13	0.98 (0.54–1.77)	0.99 (0.55–1.79)	0.99 (0.55–1.80)
Increase	21	0.97 (0.60–1.57)	0.93 (0.58–1.50)	0.93 (0.58–1.51)
Non-dense volume change per year				
Annual change by more than 10%				
Stable	121	1.00	1.00	1.00
Decrease	19	1.40 (0.86–2.29)	1.37 (0.84–2.24)	1.37 (0.84–2.23)
Increase	18	0.69 (0.41–1.14)	0.68 (0.41–1.12)	0.67 (0.41–1.11)

* Adjusted for age at FFDM (continuous) and BMI (continuous). ** Adjuster for age at FFDM (continuous), BMI (continuous), alcohol intake (never drinkers, ex-drinkers, current drinkers of ≤1, ≤2, ≤3, ≤4, >4 alcohol units). ^#^ Adjuster for age at FFDM (continuous), BMI (continuous), alcohol intake (never drinkers, ex-drinkers, current drinkers of ≤1, ≤2, ≤3, ≤4, and >4 alcohol units), Birth Index, and age at menarche.

## Data Availability

The datasets generated during and/or analysed during the current study are not publicly available due to participants privacy protection but are available from the corresponding author on reasonable request.

## Supplementary Materials

The following supporting information can be downloaded at: https://www.mdpi.com/article/10.3390/cancers15061810/s1, Figure S1: Volumetric percent density (VPD, %) distribution in consecutive full-filled digital mammograms (FFDMs) in breast cancer (BC) cases (*n* = 262) and non-cases (*n* = 5886) from the FEDRA longitudinal study. Boxes show interquartile ranges (IQR) of the distribution, horizontal lines denote median values, whiskers represent 1.5-times the IQR, dots represent outliers’ values. Figure S2: Breast dense volume (DV, cm^3^) distribution in consecutive full-filled digital mammograms (FFDMs) in breast cancer (BC) cases (*n* = 262) and non-cases (*n* = 5886) from the FEDRA longitudinal study. Boxes show interquartile ranges (IQR) of the distribution, horizontal lines denote median values, whiskers represent 1.5-times the IQR, dots represent outliers’ values. Figure S3: Breast non dense volume (NDV, cm^3^) distribution in consecutive full-filled digital mammograms (FFDMs) in breast cancer (BC) cases (*n* = 262) and non-cases (*n* = 5886) from the FEDRA longitudinal study. Boxes show interquartile ranges (IQR) of the distribution, horizontal lines denote median values, whiskers represent 1.5-times the IQR, dots represent outliers’ values.
