# Supplementary material for: The FEDRA Longitudinal Study: Repeated Volumetric Breast Density Measures and Breast Cancer Risk
Source: Cancers (Basel). 2023 Mar 16;15(6):1810. doi: 10.3390/cancers15061810 (PMC10046534; doi:10.3390/cancers15061810)
Supplement: Supplementary file 1 [file cancers-15-01810-s001.zip › cancers-2131808-supplementary.pdf]

Supplementary Materials

The FEDRA Longitudinal Study: Repeated Volumetric Breast Density Measures and Breast Cancer Risk

Giovanna Masala [1,*], Melania Assedi [2], Benedetta Bendinelli [1], Elisa Pastore [1], Maria Antonietta Gilio [3], Vincenzo Mazzalupo [3], Andrea Querci [2], Miriam Fontana [1], Giacomo Duroni [1], Luigi Facchini [2], Calogero Saieva [2], Domenico Palli [2], Daniela Ambrogetti [3] and Saverio Caini [2]

1 Clinical Epidemiology Unit, Institute for cancer research, prevention and clinical network (ISPRO), Florence, Italy
2 Cancer Risk Factors and Lifestyle Epidemiology Unit, Institute for cancer research, prevention and clinical network (ISPRO), Florence, Italy
3 Breast Cancer Screening Branch, Institute for cancer research, prevention and clinical network (ISPRO), Florence, Italy
* Correspondence: g.masala@ispro.toscana.it

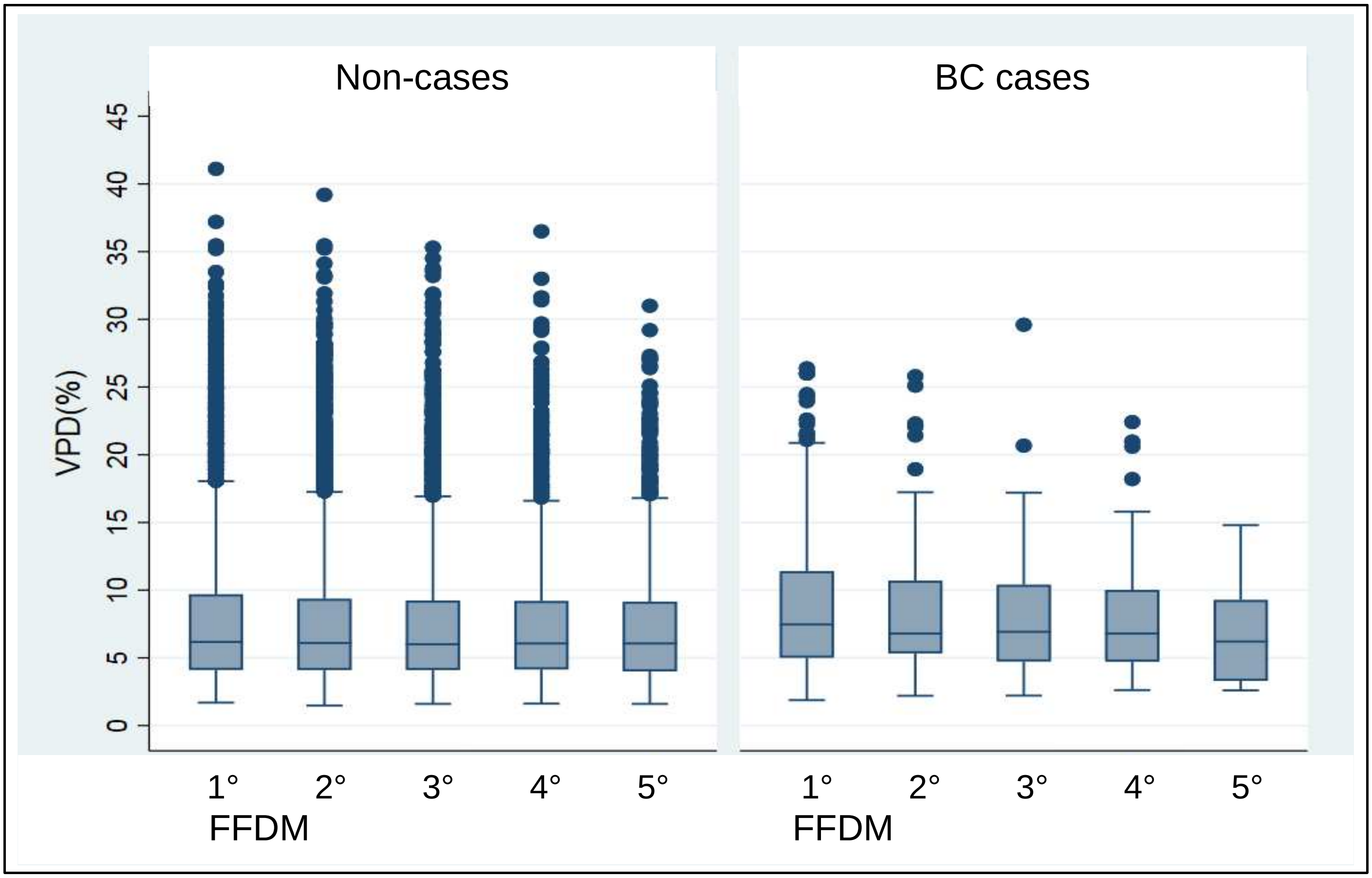

Figure S1: Volumetric percent density (VPD, %) distribution in consecutive full-filled digital mammograms (FFDMs) in breast cancer (BC) cases (n=262) and non-cases (n=5886) from the FEDRA longitudinal study. Boxes show interquartile ranges (IQR) of the distribution, horizontal lines denote median values, whiskers represents 1.5-times the IQR, dots represent outliers values.

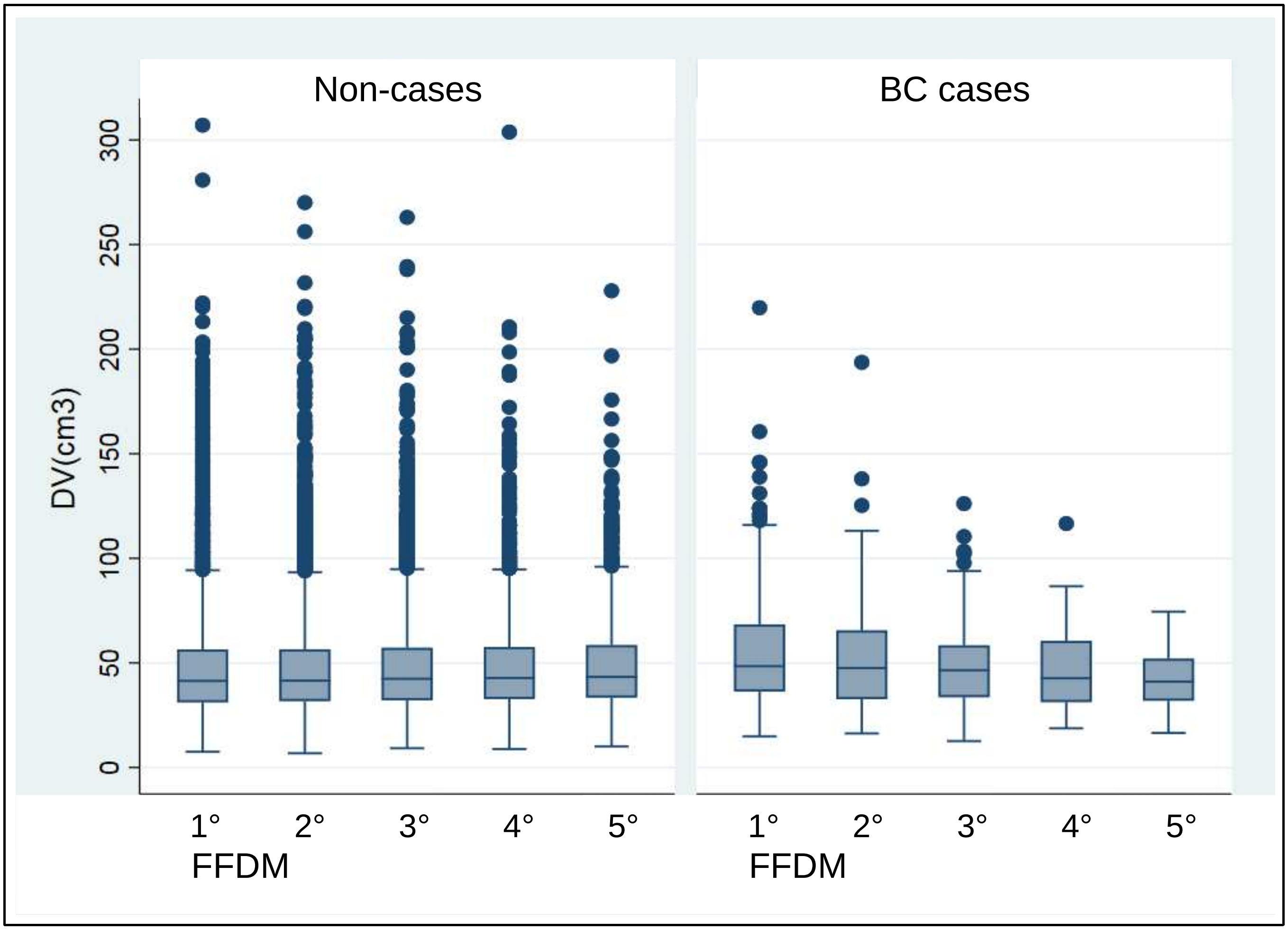

Figure S2: Breast dense volume (DV, cm^3) distribution in consecutive full-filled digital mammograms (FFDMs) in breast cancer (BC) cases (n=262) and non-cases (n=5886) from the FEDRA longitudinal study. Boxes show interquartile ranges (IQR) of the distribution, horizontal lines denote median values, whiskers represents 1.5-times the IQR, dots represent outliers values.

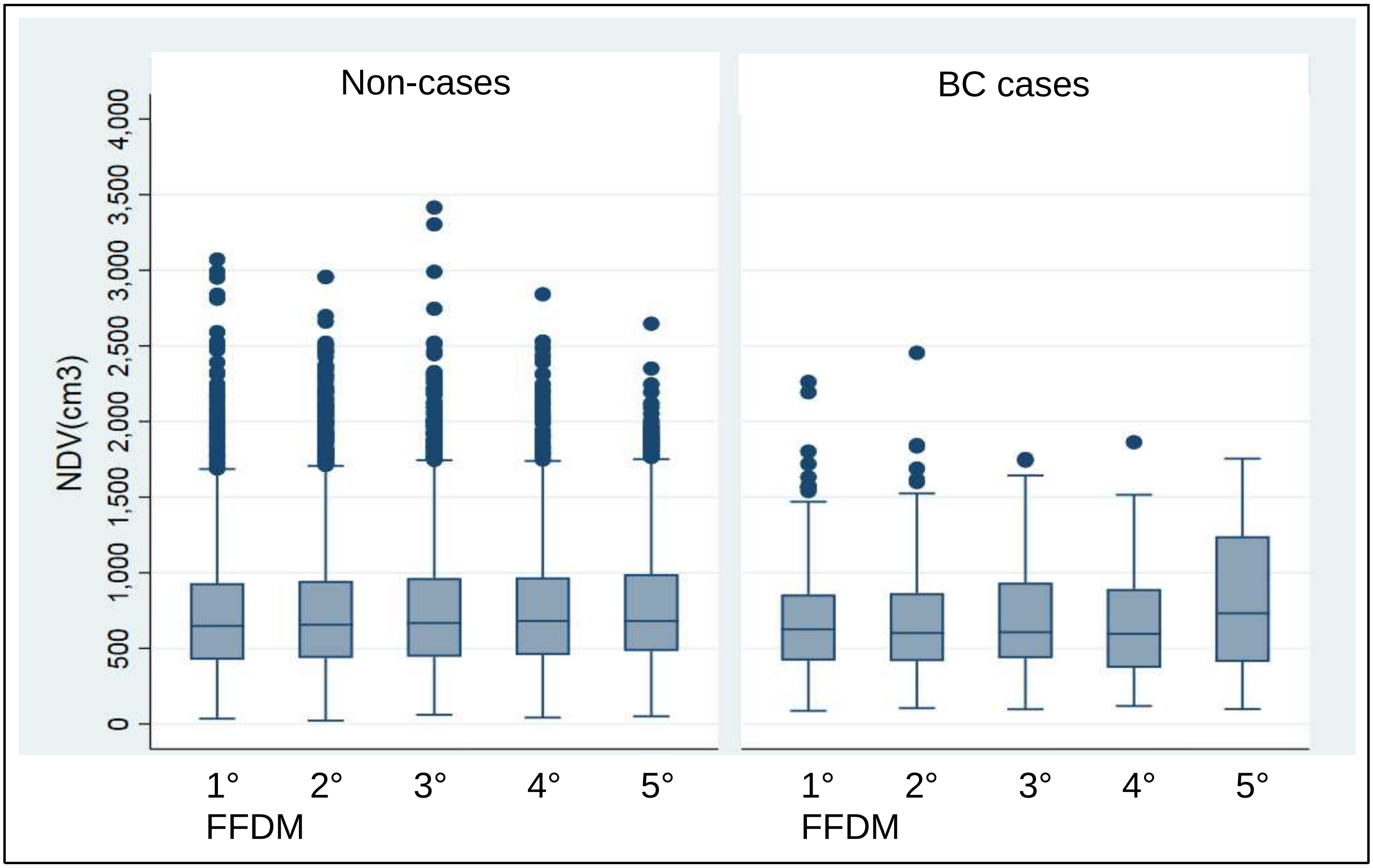

Figure S3: Breast non dense volume (NDV, cm^3) distribution in consecutive full-filled digital mammograms (FFDMs) in breast cancer (BC) cases (n=262) and non-cases (n=5886) from the FEDRA longitudinal study. Boxes show interquartile ranges (IQR) of the distribution, horizontal lines denote median values, whiskers represents 1.5-times the IQR, dots represent outliers values.
